# Associations of sitting and physical activity with grip strength and balance in mid‐life: 1970 British Cohort Study

**DOI:** 10.1111/sms.13793

**Published:** 2020-08-25

**Authors:** Rachel Cooper, Emmanuel Stamatakis, Mark Hamer

**Affiliations:** ^1^ Department of Sport and Exercise Sciences Musculoskeletal Science and Sports Medicine Research Centre Manchester Metropolitan University Manchester UK; ^2^ Faculty of Medicine and Health Charles Perkins Centre School of Health Sciences University of Sydney Sydney NSW Australia; ^3^ Institute of Sport, Exercise & Health Division of Surgery & Interventional Science Faculty of Medical Sciences University College London London UK

**Keywords:** balance, birth cohort, grip strength, physical activity, population health, sedentary, sitting

## Abstract

Strength and balance training are now recommended in many physical activity (PA) guidelines. However, it is unclear whether these recommendations are applicable to middle‐aged adults. We aimed to examine the associations of sitting and physical activity times with grip strength and standing balance performance in mid‐life. Up to 4726 participants from the 1970 British Cohort study, with data on sitting and activity (measured using a thigh‐worn accelerometer (activPAL3‐micro)), grip strength and balance times at age 46 years were included in analyses. Associations of sitting, moderate‐vigorous, and total PA times with grip strength and balance performance were tested using linear and multinomial logistic regression models, respectively. Greater time spent sitting was associated with weaker grip strength even after adjustment for potential confounders and MVPA time (fully adjusted regression coefficient: −0.51 kg (95% CI: −0.63, −0.39) per 1‐hour sitting/day). Associations of PA time with grip strength were not independent of sitting time. There was only a weak association between sitting time and balance performance but greater MVPA and total PA times were associated with higher relative risks of successfully balancing for 30 seconds with eyes closed (vs poor balance). However, these associations were fully attenuated after adjustments for covariates. In summary, among a sample of middle‐aged adults a robust association between sitting time and grip strength was observed. These findings suggest potential benefits of actively promoting less sitting alongside activities that specifically benefit muscle strength and balance performance in mid‐life to ensure that people maintain all important aspects of their physical capability as they age.

## 1 INTRODUCTION

Muscle strengthening activities and balance training are now recommended in many national and international physical activity (PA) guidelines.^1^, ^2^ This reflects growing awareness of the importance of improving and maintaining muscle strength and balance ability across life for healthy aging.^3^ Unfortunately, evidence shows that these activity recommendations have not been widely communicated nor received the attention they warrant in health promotion policies and surveillance.^4^, ^5^ To help inform strategies that aim to address this, a better understanding of the relationships of physical activity and sedentary behavior with muscle strength and balance performance at different life stages is required. When investigating these relationships, useful insights are likely to be provided by studies incorporating device‐measured (accelerometry) PA data.

Over the last two decades, the advent of affordable research‐grade wearable technology has facilitated the valid assessment and characterization of a wide range of different parameters of PA and sedentary behavior at scale and led to major advances in the study of free‐living physical activity and sedentary behavior.^6^ As sitting time was especially difficult to characterize accurately using self‐reports, the availability of accelerometry data has led to notable improvements in our understanding of the potential importance of this sedentary behavior, independent of moderate‐vigorous PA, for a range of health outcomes.^7^ While this has already prompted the inclusion of recommendations on sedentary behavior in many PA guidelines, it has been argued that this evidence base still requires development and refinement before more specific recommendations should be made.^8^

In recent years, a number of studies have examined the associations of accelerometer‐derived measures of PA and/or sedentary time with muscle strength (typically grip strength), balance ability, and other objective measures of physical capability in community‐dwelling samples (see Table S1 for summary of these studies).^9^, ^10^, ^11^, ^12^, ^13^, ^14^, ^15^, ^16^, ^17^, ^18^, ^19^, ^20^, ^21^, ^22^, ^23^, ^24^, ^25^, ^26^ While many studies report that more time spent active is associated with higher levels of physical capability, there is considerable heterogeneity in findings between studies and between different measures of physical capability. In addition, results on sedentary behavior are notably inconsistent. This may in part relate to the use of different types of accelerometer and wear positions. This is especially as hip‐ and wrist‐worn devices cannot reliably distinguish between sitting and light‐intensity activity such as standing whereby there is likely to be considerable misclassification of sedentary time in studies using these types of devices.^27^ Thigh‐worn accelerometers can overcome this as they reliably measure sitting.^28^ However, only a few existing studies have examined associations between sitting, derived from thigh‐worn accelerometry, and physical capabiclity.^14^, ^17^, ^18^, ^22^ Three of these studies had relatively small sample sizes (N = 44,^14^ 123,^18^ and 602^17^) and all had other potential limitations.

The potential limitations of these four studies are similar to those of other studies on accelerometry‐derived PA and sedentary time and objectively measured physical capability. For example, many studies have included relatively small samples (ie N < 500) and so have low statistical power.^12^, ^14^, ^16^, ^18^, ^19^, ^20^, ^21^, ^23^, ^24^, ^25^, ^26^ In addition, the majority of studies have focused on older adults, typically aged 60‐70 years and above.^9^, ^10^, ^11^, ^12^, ^14^, ^16^, ^18^, ^19^, ^20^, ^21^, ^23^, ^26^ This is justified on the basis that older adults are more likely to have muscle weakness and poor balance ability than younger adults and are therefore at greater short‐term risk of the adverse health consequences of low physical capability. However, as age‐related declines in physical capability and health status may already have precipitated declines in physical activity among older adults, reverse causality is a cause for concern in these studies. In addition, results found in older populations may not be generalizable to younger populations. This is supported by findings from an Australian study that included participants aged 36‐80 years.^17^ In this study, there was some evidence to suggest that associations between free‐living activity and physical capability were stronger in older than younger participants.

The value of studying markers of aging, such as muscle strength and balance ability, within a life course framework is now widely recognized.^29^ However, there remains a paucity of data on free‐living activity and sedentary behavior in relation to physical capability among middle‐aged adults. Our aim was to address this gap. Using data from the 1970 British Cohort Study (BCS70), a relatively large, national birth cohort study who underwent a biomedical assessment at age 46 years, we aimed to test associations of accelerometer‐derived measures of sitting and moderate‐vigorous intensity and total PA times with grip strength and standing balance performance.

## 2 MATERIALS AND METHODS

### 2.1 Study design and participants

**Figure 1:**
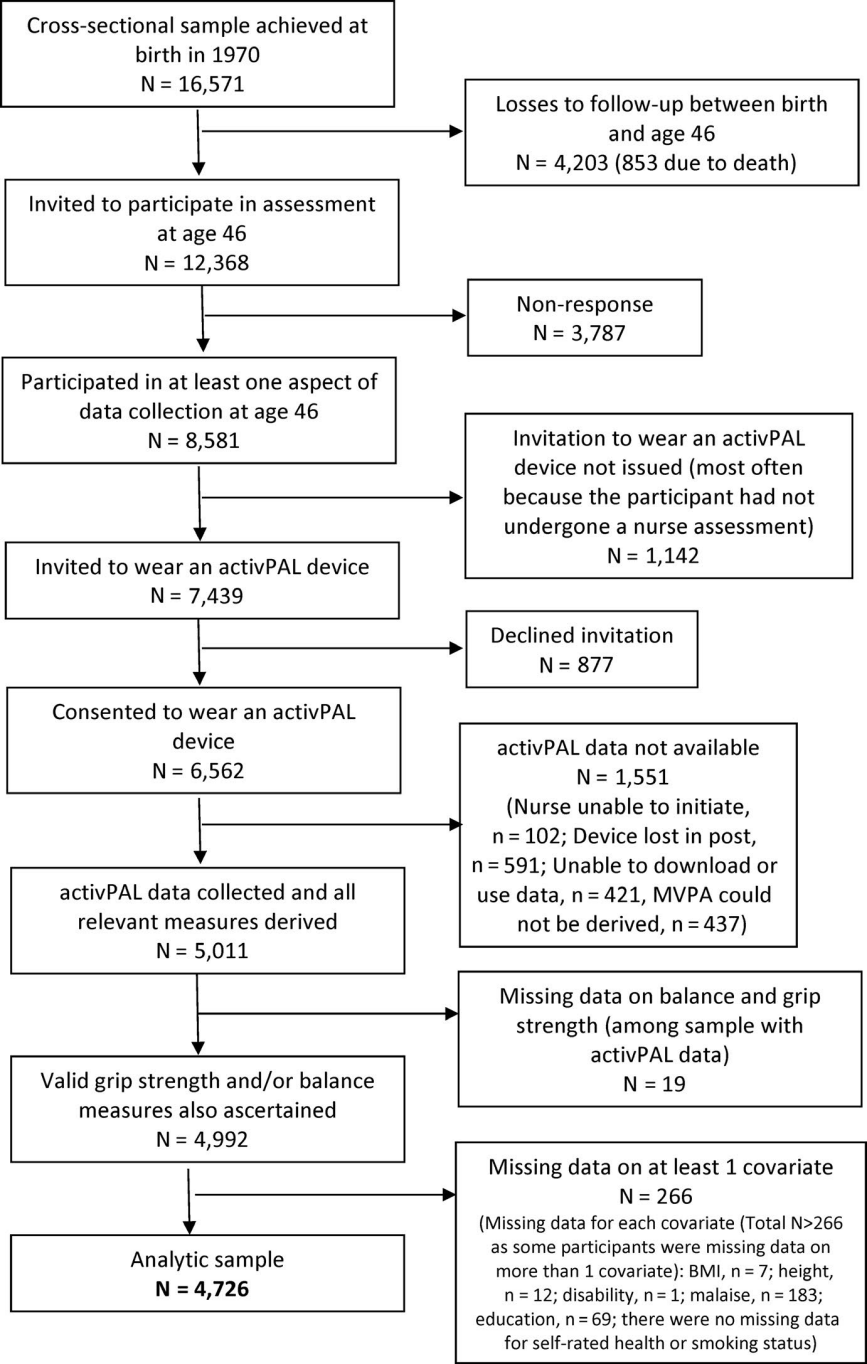
Flow diagram of participation

The BCS70 is comprised of males and females born in England, Scotland, and Wales during a single week in 1970.^30^ Study participants who were recruited at birth have been followed up across life with regular assessments throughout childhood and adulthood. During the most recent assessment in 2016‐2018, when participants were aged 46 years, a home visit was conducted which involved 50 minutes of interviews (both face‐to‐face computer‐assisted personal interview and computer‐assisted self‐completion interview) and a further 50‐minute assessment during which a trained nurse ascertained a comprehensive set of biomedical measures.^31^ Of the 16 571 males and females recruited to the original cohort, 12 368 were invited to participate in the assessment at age 46 of whom 7439 were invited to wear an accelerometer (see Figure 1 for further details). Of those 4203 not invited to participate at age 46, 853 had died, and the remainder had withdrawn, moved abroad, or were no longer contactable. Participants provided informed consent and the study received full ethical approval from NRES Committee South East Coast—Brighton & Sussex (Ref 15/LO/1446).

### 2.2 Assessment of sitting and physical activity

At the end of their biomedical assessment participants were invited to wear a thigh‐mounted accelerometer device (activPAL3 micro; PAL Technologies Ltd., Glasgow, UK)—this device uses derived information about thigh inclination and acceleration to estimate body posture (ie, sitting/lying and upright) and transition between these postures, stepping, and stepping speed (cadence). Importantly, this technique overcomes concerns raised^32^ about the face validity of hip‐ and wrist‐worn monitors to accurately capture postural sitting. The activPAL device has been validated for measuring free‐living sedentary behavior against direct observation using an automated camera.^27^, ^28^ We utilized a wear protocol previously adopted.^33^ This involved programming devices to sample at the default frequency of 20 Hz. The device was waterproofed and fitted by a trained nurse on the midline anterior aspect of the upper thigh as recommended by the manufacturer at the end of the home visit. Participants were asked to wear the device continuously for seven days, including while sleeping, bathing, swimming, and participating in any physical activity. If the device fell off or was removed before the end of the seven‐day monitoring period, participants were requested not to re‐attach it. At the end of the seven‐day monitoring period, participants were asked to remove and return their device to a central research office via the post. Data were then processed using previously validated software^33^ that uses an algorithm to isolate valid waking wear data from sleep or prolonged non‐wear. The algorithm rules have been summarized elsewhere.^34^ The first partial day was removed, and subsequent days were defined from midnight to midnight. Participants were included in the present analyses if they recorded at least one valid day during the monitoring period, defined as at least ten hours of waking wear time. In these analyses, we use three variables derived from these algorithms, time spent (hours/day): sitting, in moderate‐vigorous intensity PA (MVPA) and in total PA with a step cadence threshold ≥ 100 used to derive the MVPA measure.^35^

### 2.3 Assessment of grip strength and standing balance performance

During the biomedical assessments, nurses measured study participant's grip strength and standing balance performance using standardized protocols as follows.

Grip strength was assessed using a Smedley spring‐gauge hand‐held dynamometer. Participants were instructed to hold the device in the specified hand and then squeeze its handle as hard as they could for two seconds with the value achieved in kg recorded by the nurse before the device was reset. Participants were asked to stand without arm support to conduct the test but participants were also allowed to conduct the test with arm support and seated if required. The test was performed up to 6 times, 3 times in each hand, alternating tests between hands. The maximum grip strength (kg) achieved was used in analyses. Participants were excluded if they reported swelling or inflammation, severe pain, or a recent injury to their hands or had surgery on their hands in the last 6 months with these and other reasons for being unable to complete the assessment recorded by the nurse.

To assess standing balance performance, participants were first asked to stand on their preferred leg and raise their other foot off the ground a few inches while holding the position for as long as possible up to a maximum of 30 seconds. Participants were told that they could support themselves on a chair, table, or wall while they got into position and that they could use their arms, bend their knee, or move their body to maintain their balance during the test but that they could not move their standing foot. The nurse commenced timing as soon as the participant had raised one leg off the ground and terminated timing when balance was lost (as indicated by the raised foot touching the floor or the foot on the floor moving out of position) or 30 seconds was achieved. If a balance time with eyes open of 30 seconds was recorded, participants were then asked to repeat the test with their eyes closed. If participants felt that they would be unsafe or reported health reasons for being unable to complete the tests, this was recorded by the nurse. As balance times were highly skewed and participants who did not achieve 30s with eyes open were not asked to complete the test with eyes closed, for analytical purposes we categorized participants’ balance performance into five groups based on the time/s they achieved in the tests:<15.0s eyes open; 15.0‐29.9s eyes open; 30s eyes open and < 15.0s eyes closed; 30s eyes open and 15.0‐29.9s eyes closed; and 30s eyes open and 30s eyes closed.

### 2.4 Covariates

Potential confounders were selected a priori based on previous literature and included sex, device waking wear time, body mass index (BMI), height, self‐rated health, disability, malaise score, smoking status, and educational level, all of which were assessed at age 46. Height and weight were measured by nurses and used to calculate BMI (kg/m^2^) with missing values of BMI replaced with self‐reported data where available (n = 60). Self‐rated health was assessed by asking participants whether in general, they would say their health is excellent, very good, good, fair, or poor. To assess disability, participants were asked if they had any longstanding physical or mental health conditions or illnesses lasting or expected to last for 12 months or more and the impact of this on their daily activities. Responses to these questions were used to derive a variable categorized using the European Statistics of Income and Living Conditions (EU‐SILC) classification^36^ of none, limited to some extent, severely hampered. An assessment of mental health (emotional disturbance, well‐being, and stress) was undertaken using a 9‐item Malaise inventory from which an ordinal variable (0 (low malaise) to 9 (high malaise)) was derived. Participants were asked to report their current smoking status, categorized as never smoker, ex‐smoker, current smoker (less than daily), and current smoker (daily). New educational qualifications reported at age 46 were used to update historical data on study participant's highest educational level attained which was categorized as none, any level of qualification up to A levels (the highest qualification that can be obtained within the British school system) or their equivalents, and university degree or higher.

### 2.5 Statistical analyses

Linear and multinomial logistic regression models were used to assess the associations of sitting, MVPA, and total PA times (modeled continuously) with grip strength and standing balance performance, respectively. All models were adjusted for wear time. In initial models, we formally tested for interactions between sex and the relevant activPAL‐derived measure. Where evidence of interaction was found subsequent models were stratified by sex, otherwise sex adjustments were used. We then adjusted for height and BMI before adding all other covariates to the model. In a final set of models, we included an adjustment for MVPA time in models of sitting time, and sitting time in models of MVPA and total PA times. We tested for evidence of deviations from linearity by including a quadratic term for the relevant activPAL‐derived measure in a basic sex and wear time‐adjusted model and where evidence of this was found models were rerun with the activPAL‐derived measure categorized into fifths.

All models were run on the sample with complete data on all three activPAL‐derived measures, grip strength and/or balance performance, and covariates. As shown in Figure 1, of the 7439 participants invited to wear an accelerometer at age 46, it was possible to derive data on sitting, MVPA, and total PA times for 5011 participants and of these, 4726 also had grip strength and/or balance measures and complete data on covariates (N = 4702 for grip strength and 4644 for balance performance).

Analyses were conducted using STATA version 16.0.

### 2.6 Sensitivity analyses

To check that grip strength results were not influenced by variation between participants in their positioning during testing, the main grip strength models were rerun with adjustment for testing position (ie standing without arm support, standing with arm support, seated without arm support, or seated with arm support). To assess the possibility of reverse causation (ie low levels of physical capability leading to greater sitting time and less time active), all models were rerun after excluding participants classified as being severely hampered according to the EU‐SILC disability definition. To assess the impact of excluding participants with missing data on covariates from our main analyses, we reran sex and wear time‐adjusted models on the maximum available samples. Lastly, we repeated all analyses using a measure of self‐reported PA. The rationale being that device‐measured and self‐reported PA may capture information on different important aspects of activity.

## 3 RESULTS

Characteristics of the 4726 participants included in analyses are presented in Table 1. Men had notably higher mean grip strength than women (48.2 kg (8.8) vs 29.9 kg (5.6), *P*‐value from t test < 0.001) and also recorded better balance performance (*P*‐value from chi‐square test = 0.01). Both men and women spent an average of over 9 waking hours per day sitting and less than 1 hour per day in MVPA.

**Table 1:** Characteristics of the 1970 British Cohort study at age 46 years (sample restricted to those with complete data on grip strength or balance, activPAL measures, and covariates at age 46 (maximum N = 4726))

	Men		Women	
	N^a^	% or Mean (SD)	N^a^	% or Mean (SD)
Physical capability				
Maximum grip strength (kg)	2240^b^	48.2 (8.8)	2462^b^	29.9 (5.6)
Balance time achieved (seconds)^c^				
<15, eyes open	120	5.4	133	5.5
15‐29.9, eyes open	138	6.3	176	7.2
30 eyes open, <15 eyes closed	1302	59.0	1525	62.6
30 eyes open, 15‐29.9 eyes closed	328	14.9	310	12.7
30 eyes open and 30 eyes closed	320	14.5	292	12.0
ActivPAL‐derived measures^d^				
Sitting time (hours/day)	2244	9.5 (2.0)	2482	9.0 (1.9)
Time spent in moderate to vigorous physical activity (hours/day)	2244	0.8 (0.4)	2482	0.8 (0.4)
Time spent in physical activity (total) (hours/day)	2244	2.0 (0.7)	2482	2.0 (0.7)
Waking hours wear time (hours/day)	2244	16.0 (1.3)	2482	15.7 (1.3)
Covariates				
Body mass index (kg/m^2^)	2244	28.7 (4.9)	2482	28.1 (6.2)
Height (cm)	2244	177.0 (6.8)	2482	163.9 (6.2)
Self‐rated health
Excellent	396	17.7	514	20.7
Very good	845	37.7	915	36.9
Good	649	28.9	650	26.2
Fair	270	12.0	301	12.1
Poor	84	3.7	102	4.1
EU‐SILC disability classification
None	1964	87.5	2056	82.8
Limited to some extent	187	8.3	294	11.9
Severely hampered	93	4.1	132	5.3
Malaise score^e^				
0 (Low)	1055	47.0	863	34.8
1‐3	865	38.6	1138	45.9
4+ (High)	324	14.4	481	19.4
Smoking status				
Never smoker	1112	50.0	1264	50.9
Ex‐smoker	712	31.7	803	32.4
Current smoker (less than daily)	113	5.0	114	4.6
Current smoker (daily)	307	13.7	301	12.1
Highest educational level attained				
None	662	29.5	565	22.8
Up to A levels, or their equivalents	976	43.5	1220	49.2
Degree or higher	606	27.0	697	28.1

^a^ Total Ns lower for grip strength and standing balance times as 24 participants with complete data on all other relevant variables had missing data on grip strength but not balance times and 82 participants had missing data on balance times but not grip strength.

^b^ 93.6% of men and 90.6% of women completed the grip strength test standing without arm support.

^c^ Balance assessment consisted of a test with eyes open followed by a test with eyes closed. Only those participants who achieved the maximum time of 30s with eyes open were assessed with their eyes closed. Median balance times with eyes closed for those participants who completed this component of the balance test were 9.0s for men (N = 1950) and 7.6s for women (N = 2127).

^d^ ActivPAL monitors were worn by participants for between 1 and 8 days with 59.8% of men and 64.6% of women wearing the device for 7 days and the majority (ie, 89% of men and 91% of women) wearing them for at least 4 days.

^e^ Descriptive statistics presented as categories for brevity but the malaise score was included as an ordinal scale (0 to 9) in regression models.

More time spent sitting was associated with weaker grip strength in both men and women (Table 2), and this association was maintained after adjustment for covariates and MVPA time. Although there was evidence of deviation from linearity in a basic sex and wear time‐adjusted model (quadratic term, *P* = .001), when sitting time was modeled in fifths, this confirmed the relationship between greater time spent sitting and weaker grip strength (see Table S2).

**Table 2:** Associations of activPAL‐derived measures of sitting, moderate‐vigorous physical activity, and total physical activity times with grip strength at age 46 years (N = 4702)

Model	Differences in mean grip strength (kg) at age 46 (95% CI)			
	1	2	3	4
Sitting				
Per 1h/day increase	−0.36 (−0.47, −0.24)	−0.56 (−0.67, −0.45)	−0.46 (−0.57, −0.35)	−0.51 (−0.63, −0.39)
MVPA				
Men				
Per 1h/day increase	−1.17 (−2.01, −0.33)	−0.09 (−0.91, 0.74)	−0.52 (−1.35, 0.32)	−1.56 (−2.43, −0.68)
Women				
Per 1h/day increase	0.73 (0.19, 1.27)	1.53 (0.99, 2.08)	0.90 (0.35, 1.45)	0.34 (−0.25, 0.93)
Total PA				
Per 1h/day increase	0.60 (0.30, 0.90)	1.03 (0.74, 1.33)	0.73 (0.43, 1.02)	−0.13 (−0.52, 0.25)

Note

Model adjustments:

1: waking hours wear time and sex (where appropriate) (likelihood ratio tests of sex interaction: sitting time *P* = .15, MVPA *P* < .001, Total PA *P* = .89) (p‐values for quadratic terms: sitting time *P* = .001, MVPA men *P* = .12, women *P* = .47, Total PA *P* < .001—see Table S2 for results when modeling sitting and total PA time in fifths).

2: Model 1 plus BMI and height.

3: Model 2 plus self‐rated health, disability, malaise, smoking status, and education.

4: Model 3 plus sitting time (for models where MVPA and total PA are the main independent variable) or MVPA (where sitting time is the main independent variable).

Higher total PA time was associated with stronger grip in both sexes. This association was not explained by covariates but it was fully attenuated on adjustment for sitting time. Although there was evidence of deviation from linearity in a basic model (quadratic term, *P* < .001), when total PA time was modeled in fifths, this confirmed an association between greater total PA time and stronger grip until adjustment for sitting time (see Table S2).

Among women, findings for MVPA were consistent with those for total PA—higher MVPA times were associated with stronger grip and this was not explained by covariates but was fully attenuated on adjustment for sitting time (Table 2). Among men, there was some evidence to suggest that higher MVPA times were associated with weaker grip strength (sex interaction, *P* < .001).

In sex and wear time‐adjusted models, there was evidence that more time spent sitting was associated with lower relative risks of achieving better balance performance (vs poor performance, ie achieving < 15s with eyes open) and that greater MVPA and total PA times were associated with higher relative risks of achieving better balance performance (Table 3). However, these associations were all fully attenuated on adjustment for covariates. There was some evidence of deviation from linearity for all three activPAL‐derived measures but when they were modeled as fifths similar patterns of association were observed (see Table S3).

**Table 3:** Associations of activPAL‐derived measures of sitting, moderate‐vigorous physical activity, and total physical activity times with balance test performance at age 46 years (N = 4644)

Model	Relative risk ratios (95% CI) of achieving specified balance performance relative to the reference category of < 15s with eyes open per 1h/day increase in sitting/activity time			
	1	2	3	4
Sitting time				
15‐29.9s eyes open	0.91 (0.83, 1.00)	0.92 (0.84, 1.01)	0.96 (0.88, 1.05)	0.95 (0.86, 1.05)
< 15s eyes closed	0.88 (0.81, 0.94)	0.90 (0.83, 0.96)	0.93 (0.87, 1.00)	0.92 (0.85, 0.99)
15‐29.9s eyes closed	0.87 (0.80, 0.94)	0.90 (0.83, 0.98)	0.95 (0.87, 1.03)	0.95 (0.87, 1.04)
30s eyes closed	0.85 (0.78, 0.92)	0.90 (0.83, 0.98)	0.93 (0.85, 1.01)	0.91 (0.83, 1.00)
MVPA time				
15‐29.9s eyes open	1.36 (0.87, 2.12)	1.21 (0.77, 1.90)	0.98 (0.62, 1.53)	0.88 (0.55, 1.43)
< 15s eyes closed	1.75 (1.23, 2.48)	1.35 (0.94, 1.93)	0.93 (0.65, 1.32)	0.79 (0.54, 1.16)
15‐29.9s eyes closed	2.70 (1.84, 3.96)	1.84 (1.24, 2.73)	1.13 (0.76, 1.67)	1.01 (0.67, 1.54)
30s eyes closed	2.72 (1.85, 4.00)	1.63 (1.10, 2.43)	0.96 (0.64, 1.43)	0.81 (0.53, 1.25)
Total PA time				
15‐29.9s eyes open	1.32 (1.02, 1.72)	1.24 (0.95, 1.61)	1.10 (0.85, 1.43)	1.03 (0.74, 1.45)
< 15s eyes closed	1.46 (1.19, 1.79)	1.25 (1.01, 1.53)	1.06 (0.86, 1.30)	0.88 (0.67, 1.15)
15‐29.9s eyes closed	1.70 (1.36, 2.14)	1.35 (1.07, 1.70)	1.08 (0.86, 1.37)	0.95 (0.70, 1.30)
30s eyes closed	1.76 (1.40, 2.21)	1.31 (1.04, 1.65)	1.06 (0.84, 1.35)	0.87 (0.64, 1.19)

Note

Model adjustments:

1: waking hours wear time and sex (likelihood ratio tests of sex interaction: sitting time *P* = .66, MVPA *P* = .11, Total PA *P* = .06; likelihood ratio tests comparing model with quadratic term included with model without quadratic term: sitting time *P* < .001, MVPA *P* < .001, Total PA *P* < .001—see Table S3 for results when modeling sitting, MVPA and total PA times in fifths.

2: Model 1 plus BMI and height.

3: Model 2 plus self‐rated health, disability, malaise, smoking status, and education.

4: Model 3 plus sitting time (for models where MVPA and total PA are the main independent variable) or MVPA (where sitting time is the main independent variable).

### 3.1 Sensitivity analyses

We found no evidence of an impact of the participant's position during grip strength testing on the associations observed (see Table S4). In addition, results remained largely unchanged and conclusions remained the same: (a) after exclusion of those participants classified as severely hampered (see Tables S5 and S6) and; (b) when basic models were rerun using maximum available samples (see Tables S7 and S8). In analyses using self‐reported physical activity data, participants achieving PA guidelines had higher mean grip strength than those who reported no MVPA and there was no evidence of sex interaction (see Table S9). An association between self‐reported physical activity and balance performance was also observed and this was maintained after adjustment for covariates (see Table S10).

## 4 DISCUSSION

In a relatively large, nationally representative population of middle‐aged adults, we found evidence of a robust association between greater time spent sitting and weaker grip strength. While there was also some evidence of associations between greater total PA time (and also MVPA time among women) and stronger grip, this was not independent of sitting time. Associations of all three activPAL‐derived measures and balance performance were fully explained by adjustment for covariates including body size, physical and mental health status, and education.

There has been a surge in studies on the associations between device‐measured activity and physical capability over the past five years.^9^, ^10^, ^11^, ^12^, ^13^, ^14^, ^15^, ^16^, ^17^, ^18^, ^19^, ^20^, ^21^, ^22^, ^23^, ^24^, ^25^, ^26^ Our study adds important new insights on the nature of these associations in mid‐life and benefits from the use of a gold‐standard assessment of sitting time. This is especially important as the majority of previous studies have employed hip‐worn devices (see Table S1) which may not accurately capture sitting time.^32^ Only four studies, we identified^14^, ^17^, ^18^, ^22^ have previously used data derived from thigh‐worn devices and only one of these examined balance performance.^14^ In this study, which also included an assessment of grip strength, there was no clear evidence of associations between sedentary time and either postural stability or grip strength.^14^ However, this may have been due to limited statistical power (N = 44). In one of the other studies, the Maastricht study (aged 40‐75 years), there was only a modest association between sedentary time and grip strength and, this was not maintained after adjustment for BMI, smoking, and health status.^22^ In another of the studies, an Australian cohort, neither balance nor grip strength was assessed, and no consistent associations were found between sitting time and either of the two measures of physical capability examined (8ft Timed Up and Go and knee extensor strength).^17^

While our findings on sitting time are not fully consistent with those few studies that have used the same gold‐standard approach to measure sitting as we had, they are consistent with the finding of an association between greater sedentary time and weaker grip strength in another of the British birth cohort studies, assessed at ages 60‐64 years.^11^ This suggests that a wide range of factors, including but not limited to the use of different types of accelerometer, are likely to explain between‐study heterogeneity in findings.

Another source of between‐study heterogeneity is the use of a range of different tests to assess physical capability, with grip strength more commonly used than standing balance performance. In studies that have examined either grip strength and/or standing balance performance, there are some inconsistencies in the evidence on the strength of associations with physical activity. For example, while some studies have observed positive associations between time spent physically active and grip strength,^9^, ^11^, ^15^ another study found associations in men but not women,^10^ and other studies have found no evidence of association in either sex.^23^, ^26^ In addition, observed associations between total PA, higher intensity PA, and grip strength were not maintained after adjustment for other health behaviors and health status in one study.^22^

In studies where associations of device‐measured PA and grip strength were reported, findings suggest benefits of MVPA.^9^, ^11^, ^15^ Our finding on device‐measured MVPA times and grip strength in men, which was contradictory to the hypothesized direction of association, was therefore unexpected. In sensitivity analyses using self‐reported PA data, there were associations between greater levels of PA participation and stronger grip in both sexes. Device‐derived and self‐report data capture different aspects of PA (any bodily movement above a certain intensity threshold vs participation in purposeful, structured activities) as evidenced by a weak correlation between device‐derived and self‐report data on MVPA (*r* = 0.19) in BCS70 and differences in the daily volume of MVPA recorded using the two methods (device ~ 50 min/day vs self‐report ~ 21 min/day). As associations between self‐reported PA and grip strength were in the expected direction, this suggests that the non‐volitional component of the activity captured during free‐living assessment and classified as MVPA in the BCS70 may not be beneficial for muscle strength among middle‐aged men. In addition, as associations were in the expected direction for device‐derived total PA time, this suggests that not all PA captured by our device of benefit for strength among men in the BCS70 was classified as MVPA using the cut‐point applied. As the key strength of our selected accelerometer was the gold‐standard measurement of sitting, it is possible that this could have been at the cost of a more accurate assessment of MVPA. In another study which used the activPAL device to assess sitting, participants were asked to wear an alternative device to capture MVPA.^14^ Potential differences in the accuracy of the assessment of sitting and MVPA times may explain why associations with sitting time and grip strength were independent of MVPA but associations of total PA time (and also MVPA time among women) and grip strength were not independent of sitting time.

Accelerometers capture total volumes and patterns of movement and sedentariness but do not provide information on context. In considering the implications of our findings, specifically the association between greater sitting time and weaker grip strength, context might be important. In one study of older adults, more time spent watching television was associated with weaker grip strength while another sedentary behavior (using the internet) was not.^37^ This may be explained by the fact that older adults spend the majority of their time while sitting watching television^14^ or it might be a proxy for a particular adverse pattern of sedentary behavior (eg TV viewing may be linked to prolonged sitting without breaks). Alternatively, it could reflect a particular confounding structure that drives associations with health outcomes.^38^ Among adults of working age, such as those in the BCS70, sitting time is also likely to be influenced by the work environment. Studies to help contextualize our findings on sitting time in a middle‐aged population would therefore be informative.

Our study has a number of strengths and limitations. The main limitation was its cross‐sectional design. As a result, we cannot exclude the possibility of reverse causation (ie low physical capability driving reductions in PA and increases in sedentariness). However, as our sample were middle‐aged and therefore assessed before the onset of major age‐related declines in physical capability, we expect this to be less of a concern than in other previous studies focusing on older adults. In addition, when we excluded participants classified as severely hampered according to the EU‐SILC disability definition, conclusions remained the same. Another potential limitation is that only those participants who agreed to wear an accelerometer could be included in our analyses. Participants who declined to wear an accelerometer were more likely to be male, smokers, and report poorer health,^39^ and so our analyses were performed on a relatively healthy sub‐sample of the BCS70. This may have introduced bias and could limit the generalizability of our findings. Another potential source of bias is the exclusion of participants with missing data on covariates. However, when basic models were rerun on maximum available samples findings were the same. An additional potential limitation was the need to model balance performance in categories because of the method of balance assessment. While our approach overcame the challenge of modeling highly skewed data (eg balance times with eyes open had a large ceiling effect) and allowed us to include all participants whether they completed only the first or both balance tests in our models, these analyses will be less well powered than those of grip strength which was modeled continuously. Key strengths of our study include the well‐characterized nature of this relatively large cohort and objective assessments of physical activity, sitting, and physical capability at an age when there may be novel opportunities to intervene to prevent future age‐related declines in capability and activity participation. A focus on the two specific measures of physical capability, that is, muscle strength and balance that are targeted in national and international PA guidelines is another key strength as our findings may therefore have direct policy relevance.

### 4.1 Perspective

There has recently been an increase in the number of studies examining associations between accelerometer‐derived measures of PA, sedentariness, and physical capability. We add new insights by studying these associations in a relatively large and young sample of adults, with sitting time measured using a gold‐standard method. Our finding of a robust association between greater sitting time and weaker grip strength suggests that strategies to reduce sitting time among working age adults may help to ensure that people maintain an important aspect of their physical capability as they age. That associations of PA times with grip strength and balance performance were less consistent suggests that middle‐aged adults may not engage in sufficient levels of the specific types of activities that are now being recommended in national and international guidelines for the benefit of strength and balance. In addition, as associations between PA times and balance performance were fully explained by adjustments for covariates, this suggests that even within a relatively young and healthy population, poor health status and adverse health behaviors may already be having an adverse impact on physical activity participation and physical capability. This highlights the importance of a life course approach to the promotion of healthy aging.

## CONFLICT OF INTEREST

MH and ES have received an unrestricted grant from PAL Technologies, Scotland, UK. RC has no conflicts to disclose.

## AUTHOR CONTRIBUTIONS

The funders had no role in the study design, in the collection, analysis and interpretation of data, in writing of the report, or in the decision to submit the paper for publication. MH and RC had full access to the data and take responsibility for the integrity and accuracy of the results. MH supervised the preparation of the accelerometry dataset. MH and RC designed the concept of the study, undertook analyses, and drafted the paper. MH and ES attained funding. All authors provided intellectual input in the critical revision of the manuscript and approved the final version.

## Supporting information

Tables S1‐S10
