# Supplementary material for: Associations of sitting and physical activity with grip strength and balance in mid‐life: 1970 British Cohort Study
Source: Scand J Med Sci Sports. 2020 Aug 25;30(12):2371–81. doi: 10.1111/sms.13793 (PMC8432142; doi:10.1111/sms.13793)
Supplement: Supplementary file 1 — Tables S1‐S10 [file SMS-30-2371-s001.docx]

**Supporting Information**

**Associations of sitting and physical activity with grip strength and balance in mid-life: 1970 British Cohort study**

Rachel Cooper, Emmanuel Stamatakis and Mark Hamer

**Supporting information table 1:** Summary of studies (listed alphabetically by first author’s surname) that have investigated the associations of accelerometer-derived measures of physical activity and/or sedentary behaviour with objective measures of physical capability in community-dwelling adults

| **Ref** | **Study details**   - **Study name/s [Country]** - **Ages** - **Ns** - **% male and % female** - **inclusion/exclusion criteria** | **Type of accelerometer and measures derived from this** | **Measures of physical capability assessed** | **Covariates** | **Main findings** |
| --- | --- | --- | --- | --- | --- |
| Aggio et al, 2016^1^ | - British Regional Heart Study (cross-sectional analyses) [UK] - 70-92y - 1286 - 100% men - All surviving BRHS participants (n=3137) were invited to attend. Those confined to a wheelchair or resident in a care home (n=7) excluded (as PA assessments not possible) | Actigraph GT3x hip-worn for 7 days in 2010-12.  Variables derived: time spent sedentary and in different intensities of PA according to standard thresholds | Gait speed; Grip strength  Main outcomes: sarcopenia and sarcopenic obesity | Social class, Dr diagnosis of medical conditions, smoking, alcohol intake | - Gait speed and grip strength were positively associated with MVPA and inversely associated with sedentary time  - Sarcopenic and severely sarcopenic men were generally less physically active than the reference group  - In multinomial logistic regression models none of the PA variables were associated with risk of sarcopenia but they were associated with severe sarcopenia |
| Bann et al, 2015^2^ | - Lifestyle Interventions and Independence for Elders (LIFE) study (cross-sectional analyses of data from an RCT) [USA] - 70-89y - 1130 - ~67% women - Participants eligible for the LIFE study if they were relatively sedentary and at heightened risk of mobility disability | Actigraph GT3X hip-worn for 3-7 consecutive days except while sleeping, bathing/showering and swimming  Variables derived: total PA, light PA, higher light PA, sedentary time | Grip strength | Age, sex, ethnicity, education, clinical site, smoking, alcohol, no. of chronic conditions, history of arthritis or rheumatism and self-rated health | - Greater time spent in higher intensities of light activity was associated with stronger grip in men but not women  - No associations between lower intensities of light activity or sedentary time and grip strength |
| Cooper et al, 2015^3^ | - MRC National Survey of Health and Development (cross-sectional analyses) [UK] - 60-64y (mean 63y) - 1727 - 48% men; 52% women - All participants of the 60-64y data collection with accelerometry and physical performance data | Actiheart (captures uniaxial movement and heart rate), chest-worn for 5 days.  Variables derived: physical activity energy expenditure (PAEE), sedentary time and total volume of MVPA | Grip strength; Chair rises; Standing balance (eyes closed); TUG | Height, weight, educational level, occupational class, smoking, presence of any long-term illness, health problem or disability which limits activity, beta-blocker medication | - In sex-adjusted models, greater time spent sedentary was associated with weaker grip strength, slower chair rise and TUG speeds and shorter balance times  - In fully-adjusted models associations were still observed for grip strength and TUG speed  - Greater PAEE and MVPA associated with better performance on all tests and these associations were maintained after adjustments |
| Davis et al, 2014^4^ | - Project OPAL (Older People and Active Living) (cross-sectional analyses) [Bristol, UK] - ≥70y (mean 78.1 (5.8)) - 217 - 50% men - All participants with valid data | ActiGraph GT1M uniaxial hip-worn on a belt for all waking hours for 7 days except during bathing or if causing discomfort.  Variables derived: total PA, minutes of MVPA, light PA, very light PA and sedentary time | SPPB as a measure of lower extremity function (LEF) plus its 3 components (balance, chair rises, walking) | Height, BMI, age, sex, educational level | - In univariate analyses, higher MVPA time associated with better LEF and all 3 components while greater sedentary time was associated with worse LEF and poorer performance in all 3 components  - In models adjusted for covariates and in which MVPA and sedentary time were adjusted for each other:  - both MVPA and sedentary time remained associated with LEF, chair rises and walking  - sedentary time remained associated with balance the association with MVPA attenuated |
| Foong et al, 2016^5^ | - Part of the Tasmanian Older Adult Cohort study (Cross-sectional analyses of a longitudinal study) [Australia] - 50 to 80y (mean age: 66y, SD: 7) - 636 (valid data at either phase 2 or phase 3) - 49.2% men; 50.8% women - Random sample of adults aged 50-80 from electoral roll. Exclusions: contraindication for MRI and institutionalisation | Actigraph GT1M accelerometers worn for 7 consecutive days.  Variables derived: time spent engaged in sedentary, light, moderate and vigorous activity | Knee extension strength; Leg strength | Smoking, calorie intake, diabetes, hypertension, alcohol intake, age, sex | -No association between sedentary time and knee extension strength or leg strength  - Dose-response relationships observed between PA and knee extension strength and leg strength(with stronger effects seen at greater intensities of activity) which were maintained after adjustments |
| Gennuso et al, 2016^6^ | - No name - Cross-sectional study of older adults (Oct 2012-May 2013) [Madison, USA] - 70y (+/- 8y) - 44 - 36% men - Inclusion criteria: aged 65+, community-dwelling, able to walk unaided | ActivPAL thigh-worn device for 1 week of assessment before physical capability assessed  Variables derived: average daily sedentary behaviour, sedentary behaviour bout length, break rate, time spent in sedentary bouts of varying lengths. | Short Physical Performance Battery (SPPB); Grip strength; Long distance (400m) corridor walk; Dynamic balance testing (postural stability); SF36 | BMI, age, race, marital status, income and education assessed but not adjusted for in analyses reported | - No statistically significant relationships between sedentary behaviour and grip strength, postural stability, fall risk, the PF subscale of the SF36 or the balance and gait subscales of the SPPB.  - There were some negative associations between sedentary behaviour and the SPPB-total score, the chair stand subscale of the SPPB and 400m walk gait speed and these were more often found in men than women |
| Keevil et al, 2016^7^ | - EPIC-Norfolk (Cross-sectional analyses) [UK] - 48-92y - 8623 had capability assessed, of whom 4051 wore an accelerometer (due to missing data N varied between 3340 to 3691) - 44% men; 56% women - None reported | GT1M Actigraph hip-worn for 7 days during waking hours (except when bathing, showering or swimming).  Variables derived: sedentary and MVPA times | Grip strength; Usual walking speed (4m); Timed chair stands | Day and month, height and weight, smoking status, current wealth, alcohol intake, co-morbidity | - Men and women in top quarter of MVPA times were stronger and had faster walking and chair rise speeds and these associations were maintained after adjustment for covariates and sedentary time  - Higher sedentary time associated with lower physical capability but only very weakly even in basic models among men and these associations were attenuated after accounting for MVPA |
| Lee et al, 2015^8^ | - Sub-cohort of the Osteoarthritis Initiative (USA - multisite) (Cross-sectional analyses (2008-2010) [USA - multisite] - 49-83y - 1168 - 45% men; 55% women - Inclusion: community-dwelling older adults at elevated risk of functional loss because of the presence of knee OA, overweight or obesity or knee symptoms. For these analyses, participants had to have confirmed radiographic knee OA. | ActiGraph GT1M hip-worn during all waking hours except during water activities for 7 consecutive days  Variables derived: average daily sedentary hours, minutes of moderate to vigorous activity and % of time registered as sedentary during wear hours | 20 metre walk; Chair stand test | Age, sex, race/ethnicity, income, education, BMI, depression, comorbidities, knee symptoms, knee OA severity, prior knee injury, other lower extremity painand MVPA | - Compared with the most sedentary group, those who accumulated less sedentary time had faster walking and chair rise speeds and these associations were maintained after adjustments |
| Liao et al, 2018^9^ | - No name - Cross-sectional study of older adults (2013) [Japan] - 65-84y (mean age: 74.5y) - 281 - 62% men - Participants randomly selected from register of all 65-84y olds in the Chiba Prefecture in Matsudo City Japan. | Triaxial accelerometer (Active Style Pro HJA-350IT) hip-worn for 7 consecutive days while awake except during bathing and water activities.  Variables derived: total amounts and patterns of sedentary behaviour (total sedentary time, no. of ≥30 min sedentary bouts, no. of sedentary breaks per sedentary hour) | Grip strength; Eyes open balance test (up to 60s); 5m walk; Timed up and go (TUG) test | Age, gender, marital status, living status, educational level, employment, life circumstances, smoking, alcohol consumption, self-reported medical history (incl. hypertension, stroke, heart disease, diabetes, gout, osteoporosis, OA, RA, cancer, dementia), BMI, MVPA | - After adjusting for potential confounders and MVPA no associations were observed between total amount and patterns of sedentary behaviour and any of the physical capability measures (unadjusted results not presented)  - In sex-stratified analyses there was weak evidence of an association between duration of sedentary bouts >30min and walking and TUG test performance in women only |
| Reid et al, 2016^10^ | - 2011/2 wave of the Australian Diabetes, Obesity and Lifestyle (AusDiab3) study (Cross-sectional analyses) [Australia] - 36-80y (mean 58.1 (SD 10.0)) - 602 - 42% men; 58% women - Sub-sample of participants of wave 3 of AusDiab longitudinal study | ActivPAL3 thigh-worn for 7 days continuous wear  Variables derived - time during waking hours spent: sitting/reclining; standing; stepping (overall and MVPA) & no. of sit-stand transitions | 8ft Timed Up and Go (TUG) time; Knee extensor strength | Age, sex, marital status, housing status, income, smoking status, country of birth, work status, family history of diabetes, self-rated health, alcohol and energy intake, BMI, depressive symptoms  Tested interactions by age category (<45; 45-54; 55-64; ≥ 65y) and sex | - There were no statistically significant associations between any of the accelerometer-derived measures and TUG time  - Stepping, light stepping and MVPA stepping were all positively associated with knee extensor strength but there were no associations with sitting, prolonged sitting or standing.  -There was evidence to suggest that some associations differed by age and when analyses were rerun stratified by age, associations of stepping with TUG time were found in older age groups |
| Reid et al, 2018^11^ | - STEPS study – baseline data from a 24-week community based RCT (Cross-sectional analyses) [Australia] - 65-84y (mean 70.9 (SD 4.2)) - 123 - 37% men; 63% women - Healthy community-dwelling men and women recruited via local newspaper and magazine adverts, word of mouth, flyer distribution and presentations at local community centres. Range of exclusion criteria applied including regular participation in moderate intensity PA, BMI>40 | ActivPAL3 thigh-worn for 7 days continuous wear  Variables derived - time spent sitting and stepping & no. of breaks in sitting time (sit-stand transitions). All measures standardised for waking wear time | Lower limb strength (knee extensor strength and one-repetition maximum bilateral leg press); dynamic standing balance and mobility (four square step test); chair rise test (30 second sit to stand); timed up and go with dual task; gait speed (4m) | Factors adjusted for: age, sex, ethnicity, marital status, total number of medications taken, current smoking status, serum 25-hydroxyvitamin D, stepping time, total body fat mass  Other outcomes: body composition (assessed using DXA) and inflammatory markers | - In models adjusted for age and sex, greater time spent sitting was associated with poorer performance in the four square step and chair rise tests  - More breaks in sitting time were associated with lower limb strength (as indicated by leg press) in a basic model but this was attenuated in a fully adjusted model  - There were no associations between sitting time or breaks in sitting and knee extensor strength, timed up and go with dual task test or gait speed |
| Rosenberg et al, 2016^12^ | - Cross-sectional analyses of baseline measures from a physical activity trial in 11 retirement communities [San Diego County, USA] - 65y+ (mean age: 83.6y) - 307 - 27.7% men - Eligibility criteria for the trial: age 65+;ability to speak and read English; ability to complete written assessments; no history of falls resulting in hospitalisation in the last 12 months; ability to walk 20m without human assistance; completion of TUG in <30s; ability to read survey questions; completion of a post-consent comprehension test | ActiGraph GT3X+ hip-worn for 6 days. Worn at all times except when sleeping, showering or swimming  Variables derived: sedentary and MVPA times | 400m walk test; Short Physical Performance Battery (SPPB) and its individual components | Other outcomes: mental health (CES-D-10); cognitive function; physical health (pain interference and sleep disturbance); blood pressure | - Greater sedentary time associated with poorer performance in 400m walk test and worse SPPB scores. |
| Santos et al, 2012^13^ | - No name - Cross-sectional study [Portugal] - 65-103y (mean 74.3 (SD 6.6)) - 312 - 38% men; 63% women - Representative sample of non-institutionalised Portuguese older adults, 65 or older, selected by means of a proportionate stratified random sampling. Inclusion criteria were that participants had to be independent in physical functioning (i.e. were able to perform all basic and instrumental ADLs) | Actigraph GT1M hip-worn for 4 consecutive days (incl 2 week days and 2 weekend days)  Variables derived, mean time per day: sedentary, light PA, moderate PA, vigorous PA, MVPA and total PA intensity | ‘Functional fitness’ – Senior Fitness Test battery devised by Rikli & Jones (incl. 30s chair stands, arm curl, chair sit and reach, back scratch, 8-foot up and go, 6 minute walk test – scores on each test standardised and summed) | Age, gender, accelerometer register, BMI | - Greater sedentary time was associated with a lower functional fitness score and this was maintained after adjustment for MVPA and potential confounders  - Greater MVPA time was associated with a higher functional fitness score independent of sedentary time |
| Sardinha et al, 2015^14^ | - No name - Cross-sectional study [Portugal] - 65-94y (mean 73.3 (SD 5.9)) - 215 - 40% men; 60% women - Representative sample of non-institutionalised Portuguese older adults, 65 or older, selected by means of a proportionate stratified random sampling. | Actigraph GT1M hip-worn for 4 consecutive days (incl 2 week days and 2 weekend days)  Variables derived: MVPA time, sedentary time and breaks in sedentary time (any interruption in sedentary time in which the accelerometer count rose up to or above 100 counts/min) | ‘Functional fitness’ – Senior Fitness Test battery (see details above (Santos et al)) | Age, sex, BMI, educational attainment, physical independence, medical history of hypertension, elevated cholesterol and glycaemia, current medication, presence of any long-standing condition (incl. diabetes, asthma, cancer, cardiac disease), smoking | - More breaks in sedentary time were associated with better arm curl and chair stand test performance and a higher functional fitness score and this was maintained after adjusting for MVPA and sedentary times  - The association between more breaks in sedentary time and composite functional test score was also maintained after controlling for covariates  - Greater MVPA time was associated with a higher functional fitness score independent of sedentary time and breaks in sedentary time |
| Semanik et al, 2015^15^ | - Sub-cohort of the Osteoarthritis Initiative (Longitudinal (2008-2012)) [USA – multisite] - 49-83y (mean 64.8 (SD 9.0)) - 1659 - 45.3% men; 54.7% women - Inclusion: community-dwelling older adults at elevated risk of functional loss because of the presence of knee OA, overweight or obesity or knee symptoms. Excluded: people with incomplete follow-up and missing data | ActiGraph GT1M hip-worn during all waking hours except during water activities for 7 consecutive days  Activity and sedentary behaviour assessed at baseline (2008-2010).  Variables derived: average daily sedentary time (hours) and daily % of time registered as sedentary during wear hours | Changes in gait speed and chair stands modelled as a score (differences between baseline and 2y follow-up) | Baseline function, age, sex, race/ethnicity, income, education, obesity, depression, comorbidities, knee symptoms, knee OA severity, prior knee injury, other lower extremity pain, smoking and MVPA | - Greater time spent sedentary at baseline was associated with larger declines in both gait speed and chair rise performance over 2 years  - These associations were maintained after adjustments for covariates including MVPA |
| van der Velde et al, 2017^16^ | - Maastricht Study (cross-sectional analyses of a convenience sample of the 1^st^ participants to complete the baseline survey between Nov 2010 and Sept 2013) [Netherlands] - 40-75y (mean 59.7 (SD 8.2)) - 1,932 - 51.4% men; 48.6% women - Participants (aged 40 to 75 and living in the southern part of the Netherlands) recruited via mass media campaigns. Of first 3451 participants, exclusions were for missing accelerometer data, missing physical function data and missing covariates | activPAL3 thigh-worn for 8 consecutive days without removal at any time  Variables derived: sedentary time (sitting or lying) (ST) (h), no. of sedentary breaks (n), prolonged (>=30 min) sedentary bouts (n), total PA, time spent in higher intensity PA (h) | Distance walked at fast pace during 6 min walk test [6MWD]; Timed chair rise stands; Grip strength; Elbow flexion and knee extension strength | Age, sex, educational level, smoking, alcohol, CVD history, self-reported physical functioning (SF36), self-rated health status, BMI, type 2 diabetes | - There were modest associations between sedentary time (and also sedentary breaks and sedentary bout duration) and all 5 outcomes in expected directions in basic age, sex and wear time adjusted models.  - After adjustment for all covariates only the associations of sedentary time with 6MWD and elbow flexion strength were maintained  - Greater total PA time was associated with all outcomes in expected directions in basic models and all except the association with grip strength was maintained in fully-adjusted models |
| Westbury et al, 2018^17^ | - Hertfordshire Sarcopenia Study (cross-sectional analyses) [UK] - 74-84y (mean 79y (SD 3)) - 131 - 24% men; 76% women - Exclusions: concurrent use of anticoagulant medication, neuromuscular comorbidity or diabetes | GENEactiv worn on non-dominant wrist for 7 days.  Variables derived: mean daily acceleration and min/day spent in non-sedentary and MVPA | Grip strength; Gait speed (customary pace over 3m) | Gender, age, occupational class, height, weight-for-height residual, smoking, alcohol | - Greater acceleration and time spent in PA (namely time non-sedentary rather than MVPA) was associated with faster walking speed and these associations were maintained after adjustments for covariates  - PA times and acceleration were not associated with grip strength |
| Willoughby and Copeland, 2015^18^ | - No name – Cross-sectional study [Canada] - 50-67y (mean 56.6y (SD 4.1)) - 49 - 100% women - Female participants had to be 50-70y old and engaged in full time employment. Participants were screened for contraindications and excluded if they had any unstable health condition, acute neurological or musculoskeletal injury or physical limitation that precluded them from completing the testing | ActiGraph GT3X hip-worn for 7 days during waking hours except when engaged in aquatic activity.  Variables derived: % of time spent sedentary and in MVPA, no. of sedentary breaks | Leg strength (peak torque of knee extensors and flexors); Postural stability (computerized dynamic posturography and a composite equilibrium score) – tested on return to lab at end of 7 day activity monitoring period | Age, BMI | - In bivariate analyses, greater sedentary time was associated with poorer performance in all tests and greater MVPA time was associated with better performance.  - After adjustment for MVPA time, associations of sedentary time with the outcomes were attenuated while associations of MVPA time with the outcomes were maintained after adjustment for sedentary time |
| Wu et al, 2017^19^ | - No name – Cross-sectional study [Australia] - 36-57y (mean 50y (SD 5)) - 309 - 100% women - Participants were from a 10y follow-up of an RCT conducted in 2000 in Southern Tasmania. | Actigraph GT1M hip-worn for 7 consecutive days  Variables derived: time spent sedentary and in total, light and MVPA | Lower limb muscle strength (LMS); Timed up and go (TUG) test; Functional reach test; lateral reach test; Step test | Age, weight, height, menopausal status, calcium intake, vitamin D levels, history of fracture | - Greater volume of total PA (and time spent in MVPA but not light PA) associated with stronger lower limbs and better TUG and step test performance and the associations with leg strength and TUG were maintained after adjustment for confounders.  - Light PA and sedentary time were not associated with any of the physical capability outcomes (except for an association of sedentary time and TUG time which was attenuated after adjustment for MVPA) |
| Yasunaga et al, 2017^20^ | - No name - Cross-sectional study [Japan] - 65-84y (mean 74.4y (SD 5.2)) - 287 - 63% men, 37% women - Participants were drawn from a larger epidemiological study conducted in Matsudo City, Chiba Prefecture | Triaxial accelerometer (Active Style Pro HJA-350IT) hip-worn for 7 consecutive days while awake except during bathing and water activities  Single-activity, partition and isotemporal models used to examine associations of sedentary behaviour, light and MVPA with each outcome | Grip strength; Eyes open balance test (up to 60s); 11m walk (usual and maximum gait speed); Timed up and go (TUG) test | Age, gender, BMI, no. of past illnesses, complications and comorbidity, smoking, alcohol, living arrangement, highest educational attainment | -When examining each PA parameter in separate models: greater sedentary time was not associated with grip strength or balance but was associated with slower gait speeds and longer TUG times; there were no associations between LPA and any of the outcomes; greater MVPA time was associated with all outcomes except grip strength  - In partition models, MVPA was associated with all outcomes except grip strength but there were no associations for sedentary behaviour or LPA  - In isotemporal models, replacing sedentary or LPA time with MVPA was associated with better physical capability |

**Supporting information table 2:** Associations of activPAL-derived measures of sitting and total physical activity times, modelled in fifths, with grip strength at age 46 (N=4,702)

|  | **Difference in mean grip strength (kg) at age 46 (95% CI)** | | | |
| --- | --- | --- | --- | --- |
| **Model:** | **1** | **2** | **3** | **4** |
| **Sitting time (h/day)**  Lowest: 1 (< 7.57)  2 (7.57 – 8.79)  3 (8.80 – 9.79)  4 (9.80 – 10.91)  Highest: 5 (> 10.91) | 0  -0.20 (-0.86, 0.46)  -0.53 (-1.19, 0.13)  -0.86 (-1.53, -0.19)  -1.97 (-2.67, -1.28) | 0  -0.45 (-1.08, 0.18)  -0.99 (-1.62, -0.36)  -1.58 (-2.22, -0.94)  -3.12 (-3.79, -2.45) | 0  -0.31 (-0.93, 0.31)  -0.85 (-1.48, -0.22)  -1.29 (-1.93, -0.66)  -2.63 (-3.30, -1.96) | 0  -0.38 (-1.01, 0.25)  -0.96 (-1.59, -0.32)  -1.46 (-2.12, -0.81)  -2.88 (-3.59, -2.17) |
| **Total PA time (h/day)**  Lowest: 1 (< 1.40)  2 (1.40 – 1.74)  3 (1.75 – 2.08)  4 (2.09 – 2.52)  Highest: 5 (> 2.52) | 0  0.95 (0.28, 1.61)  1.66 (1.00, 2.33)  1.33 (0.66, 1.99)  1.47 (0.80, 2.14) | 0  1.30 (0.66, 1.94)  2.03 (1.39, 2.68)  2.10 (1.45, 2.75)  2.32 (1.67, 2.98) | 0  0.98 (0.35, 1.62)  1.59 (0.95, 2.24)  1.57 (0.92, 2.22)  1.72 (1.06, 2.39) | 0  0.52 (-0.13, 1.17)  0.85 (0.16, 1.54)  0.53 (-0.21, 1.26)  0.27 (-0.55, 1.08) |

Model adjustments:

1: waking hours wear time and sex

2: Model 1 plus BMI and height

3: Model 2 plus self-rated health, disability, malaise, smoking status and education

4: Model 3 plus sitting time in fifths (where total PA is the main independent variable) or MVPA (where sitting time is the main independent variable)

**Supporting information table 3:** Associations of activPAL-derived measures of sitting and total physical activity times, modelled in fifths, with standing balance test performance at age 46 (N=4,644)

|  | **Relative risk ratios (95% CI) of achieving specified balance performance relative to the reference category of < 15s with eyes open when comparing fifths of sitting/activity time** | | | |
| --- | --- | --- | --- | --- |
| **Categories of balance performance** | 15–29.9s eyes open | <15s eyes closed | 15–29.9s eyes closed | 30s eyes closed |
| **Sitting time (h/day)**  **Model 1**  Lowest: 1 (< 7.57)  2 (7.57 – 8.79)  3 (8.80 – 9.79)  4 (9.80 – 10.91)  Highest: 5 (> 10.91) | 1.00  1.38 (0.79, 2.38)  1.08 (0.62, 1.89)  0.62 (0.36, 1.07)  0.80 (0.47, 1.38) | 1.00  1.14 (0.74, 1.78)  1.05 (0.68, 1.63)  0.66 (0.44, 0.99)  0.62 (0.41, 0.94) | 1.00  1.15 (0.70, 1.89)  1.29 (0.79, 2.09)  0.70 (0.44, 1.10)  0.61 (0.38, 0.99) | 1.00  1.39 (0.85, 2.28)  1.26 (0.77, 2.06)  0.68 (0.43, 1.09)  0.47 (0.28, 0.76) |
| **Model 2**  Lowest: 1 (< 7.57)  2 (7.57 – 8.79)  3 (8.80 – 9.79)  4 (9.80 – 10.91)  Highest: 5 (> 10.91) | 1.00  1.41 (0.81, 2.45)  1.11 (0.64, 1.95)  0.63 (0.37, 1.09)  0.84 (0.49, 1.46) | 1.00  1.19 (0.76, 1.85)  1.11 (0.71, 1.72)  0.67 (0.45, 1.01)  0.68 (0.44, 1.04) | 1.00  1.22 (0.74, 2.02)  1.43 (0.87, 2.34)  0.74 (0.46, 1.18)  0.74 (0.45, 1.22) | 1.00  1.52 (0.92, 2.51)  1.50 (0.91, 2.48)  0.79 (0.49, 1.27)  0.64 (0.38, 1.06) |
| **Model 3**  Lowest: 1 (< 7.57)  2 (7.57 – 8.79)  3 (8.80 – 9.79)  4 (9.80 – 10.91)  Highest: 5 (> 10.91) | 1.00  1.47 (0.85, 2.56)  1.22 (0.69, 2.14)  0.71 (0.41, 1.23)  1.00 (0.57, 1.74) | 1.00  1.20 (0.77, 1.88)  1.15 (0.73, 1.80)  0.72 (0.48, 1.09)  0.80 (0.52, 1.24) | 1.00  1.23 (0.74, 2.05)  1.45 (0.88, 2.39)  0.78 (0.48, 1.26)  0.89 (0.54, 1.48) | 1.00  1.47 (0.88, 2.44)  1.41 (0.85, 2.34)  0.76 (0.47, 1.23)  0.71 (0.42, 1.20) |
| **Model 4**  Lowest: 1 (< 7.57)  2 (7.57 – 8.79)  3 (8.80 – 9.79)  4 (9.80 – 10.91)  Highest: 5 (> 10.91) | 1.00  1.45 (0.83, 2.53)  1.19 (0.68, 2.10)  0.69 (0.39, 1.20)  0.95 (0.53, 1.71) | 1.00  1.17 (0.75, 1.84)  1.10 (0.70, 1.74)  0.68 (0.44, 1.04)  0.73 (0.46, 1.16) | 1.00  1.24 (0.74, 2.06)  1.46 (0.88, 2.43)  0.79 (0.48, 1.29)  0.91 (0.53, 1.55) | 1.00  1.43 (0.86, 2.38)  1.35 (0.81, 2.26)  0.71 (0.43, 1.17)  0.65 (0.37, 1.12) |
|  |  |  |  |  |
| **MVPA (h/day)**  **Model 1**  Lowest: 1 (< 0.49)  2 (0.49 – 0.69)  3 (0.70 – 0.88)  4 (0.89 – 1.15)  Highest: 5 (> 1.15) | 1.00  1.11 (0.68, 1.81)  1.52 (0.92, 2.54)  1.15 (0.69, 1.93)  1.01 (0.60, 1.70) | 1.00  1.50 (1.03, 2.18)  1.88 (1.25, 2.82)  1.75 (1.17, 2.61)  1.62 (1.09, 2.41) | 1.00  2.10 (1.34, 3.28)  2.65 (1.64, 4.27)  2.88 (1.80, 4.59)  2.91 (1.83, 4.63) | 1.00  1.74 (1.10, 2.74)  2.30 (1.41, 3.73)  3.20 (2.01, 5.11)  3.10 (1.95, 4.94) |
| **Model 2**  Lowest: 1 (< 0.49)  2 (0.49 – 0.69)  3 (0.70 – 0.88)  4 (0.89 – 1.15)  Highest: 5 (> 1.15) | 1.00  1.07 (0.66, 1.74)  1.40 (0.84, 2.35)  1.04 (0.61, 1.76)  0.88 (0.51, 1.51) | 1.00  1.37 (0.94, 2.00)  1.59 (1.05, 2.41)  1.41 (0.94, 2.13)  1.21 (0.79, 1.83) | 1.00  1.81 (1.15, 2.86)  2.06 (1.26, 3.36)  2.06 (1.27, 3.33)  1.84 (1.13, 2.98) | 1.00  1.43 (0.89, 2.28)  1.64 (0.99, 2.69)  2.03 (1.25, 3.29)  1.66 (1.02, 2.70) |
| **Model 3**  Lowest: 1 (< 0.49)  2 (0.49 – 0.69)  3 (0.70 – 0.88)  4 (0.89 – 1.15)  Highest: 5 (> 1.15) | 1.00  0.94 (0.57, 1.54)  1.21 (0.71, 2.05)  0.87 (0.50, 1.49)  0.71 (0.41, 1.24) | 1.00  1.10 (0.75, 1.62)  1.21 (0.79, 1.85)  0.99 (0.65, 1.51)  0.82 (0.53, 1.26) | 1.00  1.35 (0.84, 2.15)  1.39 (0.84, 2.29)  1.23 (0.75, 2.02)  1.06 (0.64, 1.76) | 1.00  1.02 (0.63, 1.66)  1.05 (0.63, 1.76)  1.17 (0.71, 1.93)  0.89 (0.54, 1.49) |
| **Model 4**  Lowest: 1 (< 0.49)  2 (0.49 – 0.69)  3 (0.70 – 0.88)  4 (0.89 – 1.15)  Highest: 5 (> 1.15) | 1.00  0.89 (0.54, 1.48)  1.11 (0.65, 1.91)  0.78 (0.44, 1.37)  0.62 (0.34, 1.12) | 1.00  1.03 (0.69, 1.53)  1.09 (0.70, 1.68)  0.86 (0.55, 1.34)  0.68 (0.42, 1.08) | 1.00  1.28 (0.80, 2.06)  1.29 (0.77, 2.15)  1.11 (0.66, 1.87)  0.93 (0.54, 1.59) | 1.00  0.96 (0.59, 1.56)  0.95 (0.56, 1.61)  1.03 (0.61, 1.73)  0.75 (0.43, 1.29) |
|  |  |  |  |  |
| **Total PA time (h/day)**  **Model 1**  Lowest: 1 (< 1.40)  2 (1.40 – 1.74)  3 (1.75 – 2.08)  4 (2.09 – 2.52)  Highest: 5 (> 2.52) | 1.00  1.54 (0.94, 2.52)  1.59 (0.95, 2.66)  1.37 (0.81, 2.31)  1.43 (0.87, 2.37) | 1.00  1.87 (1.28, 2.74)  2.00 (1.34, 2.98)  2.10 (1.40, 3.13)  1.75 (1.19, 2.59) | 1.00  2.37 (1.51, 3.72)  2.94 (1.85, 4.67)  2.68 (1.68, 4.27)  2.45 (1.55, 3.85) | 1.00  2.25 (1.41, 3.58)  3.36 (2.09, 5.38)  3.75 (2.34, 6.00)  2.80 (1.76, 4.45) |
| **Model 2**  Lowest: 1 (< 1.40)  2 (1.40 – 1.74)  3 (1.75 – 2.08)  4 (2.09 – 2.52)  Highest: 5 (> 2.52) | 1.00  1.47 (0.89, 2.41)  1.50 (0.89, 2.52)  1.25 (0.73, 2.14)  1.29 (0.77, 2.18) | 1.00  1.65 (1.12, 2.43)  1.70 (1.13, 2.55)  1.67 (1.11, 2.52)  1.32 (0.89, 1.98) | 1.00  1.95 (1.23, 3.08)  2.32 (1.45, 3.71)  1.87 (1.16, 3.02)  1.57 (0.98, 2.51) | 1.00  1.77 (1.10, 2.86)  2.53 (1.56, 4.11)  2.37 (1.46, 3.85)  1.58 (0.98, 2.56) |
| **Model 3**  Lowest: 1 (< 1.40)  2 (1.40 – 1.74)  3 (1.75 – 2.08)  4 (2.09 – 2.52)  Highest: 5 (> 2.52) | 1.00  1.33 (0.80, 2.20)  1.36 (0.80, 2.29)  1.08 (0.62, 1.86)  1.09 (0.64, 1.86) | 1.00  1.38 (0.93, 2.06)  1.42 (0.94, 2.15)  1.29 (0.84, 1.96)  1.00 (0.66, 1.52) | 1.00  1.51 (0.95, 2.42)  1.77 (1.09, 2.87)  1.28 (0.78, 2.10)  1.05 (0.64, 1.71) | 1.00  1.34 (0.82, 2.19)  1.88 (1.15, 3.09)  1.60 (0.97, 2.65)  1.06 (0.64, 1.74) |
| **Model 4**  Lowest: 1 (< 1.40)  2 (1.40 – 1.74)  3 (1.75 – 2.08)  4 (2.09 – 2.52)  Highest: 5 (> 2.52) | 1.00  1.24 (0.73, 2.09)  1.22 (0.69, 2.17)  0.94 (0.50, 1.75)  0.90 (0.46, 1.79) | 1.00  1.21 (0.80, 1.83)  1.16 (0.74, 1.83)  0.99 (0.61, 1.62)  0.70 (0.41, 1.19) | 1.00  1.36 (0.83, 2.21)  1.51 (0.89, 2.56)  1.04 (0.59, 1.83)  0.78 (0.42, 1.46) | 1.00  1.19 (0.71, 1.97)  1.57 (0.92, 2.70)  1.26 (0.71, 2.24)  0.76 (0.40, 0.98) |

Model adjustments:

1: waking hours wear time and sex

2: Model 1 plus BMI and height

3: Model 2 plus self-rated health, disability, malaise, smoking status and education

4: Model 3 plus sitting time (where total PA is the main independent variable) or MVPA (where sitting time is the main independent variable)

**Supporting information table 4:** Associations of activPAL-derived measures of sitting, moderate-vigorous physical activity and total physical activity times with grip strength at age 46 with additional adjustment for participant’s position during grip strength testing (N=4,702)

|  | **Difference in mean grip strength (kg) at age 46 (95% CI)** | | | |
| --- | --- | --- | --- | --- |
| **Model:** | **1** | **2** | **3** | **4** |
| **Sitting**  per 1h/day increase | -0.36 (-0.47, -0.24) | -0.56 (-0.67, -0.45) | -0.46 (-0.57, -0.35) | -0.51 (-0.63, -0.39) |
| **MVPA**  *Men*  per 1h/day increase  *Women*  per 1h/day increase | -1.16 (-2.01, -0.32)  0.72 (0.18, 1.26) | -0.09 (-0.91, 0.74)  1.52 (0.98, 2.06) | -0.51 (-1.34, 0.32)  0.90 (0.34, 1.45) | -1.55 (-2.43, -0.67)  0.34 (-0.25, 0.93) |
| **Total PA**  per 1h/day increase | 0.60 (0.30, 0.90) | 1.03 (0.73, 1.32) | 0.72 (0.43, 1.02) | -0.14 (-0.53, 0.25) |

Model adjustments:

1: waking hours wear time, position during testing (standing without arm support (92%), standing with arm support (2%), seated without arm support (5%) or seated with arm support (1%)) and sex (where appropriate)

2: Model 1 plus BMI and height

3: Model 2 plus self-rated health, disability, malaise, smoking status and education

4: Model 3 plus sitting time (for models where MVPA and total PA are the main independent variable) or MVPA (where sitting time is the main independent variable)

**Supporting information table 5:** Associations of activPAL-derived measures of sitting, moderate-vigorous physical activity and total physical activity times with grip strength at age 46 after exclusion of 222 participants with a EU-SILC disability classification of severely hampered (N=4,480)

|  | **Difference in mean grip strength (kg) at age 46 (95% CI)** | | | |
| --- | --- | --- | --- | --- |
| **Model:** | **1** | **2** | **3** | **4** |
| **Sitting**  per 1h/day increase | -0.28 (-0.39, -0.16) | -0.48 (-0.59, -0.37) | -0.44 (-0.56, -0.33) | -0.50 (-0.62, -0.38) |
| **MVPA**  *Men*  per 1h/day increase  *Women*  per 1h/day increase | -1.69 (-2.54, -0.84)  0.36 (-0.19, 0.91) | -0.54 (-1.37, 0.29)  1.17 (0.62, 1.72) | -0.69 (-1.52, 0.14)  0.87 (0.32, 1.43) | -1.65 (-2.52, -0.78)  0.32 (-0.28, 0.91) |
| **Total PA**  per 1h/day increase | 0.35 (0.05, 0.66) | 0.81 (0.51, 1.10) | 0.66 (0.36, 0.96) | -0.19 (-0.58, 0.20) |

Model adjustments:

1: waking hours wear time and sex (where appropriate)

2: Model 1 plus BMI and height

3: Model 2 plus self-rated health, disability, malaise, smoking status and education

4: Model 3 plus sitting time (for models where MVPA and total PA are the main independent variable) or MVPA (where sitting time is the main independent variable)

**Supporting information table 6:** Associations of activPAL-derived measures of sitting, moderate-vigorous physical activity and total physical activity times with balance test performance at age 46 years after exclusion of 185 participants with a EU-SILC disability classification of severely hampered (N=4,459)

|  | **Relative risk ratios (95% CI) of achieving specified balance performance relative to the reference category of < 15s with eyes open per 1h/day increase in sitting/activity time** | | | |
| --- | --- | --- | --- | --- |
| **Model:** | **1** | **2** | **3** | **4** |
| **Sitting time**  15 – 29.9s eyes open  < 15s eyes closed  15 – 29.9s eyes closed  30s eyes closed | 0.93 (0.85, 1.03)  0.90 (0.83, 0.98)  0.90 (0.82, 0.98)  0.88 (0.80, 0.96) | 0.95 (0.86, 1.05)  0.93 (0.86, 1.00)  0.94 (0.86, 1.02)  0.94 (0.86, 1.02) | 0.97 (0.88, 1.06)  0.94 (0.87, 1.01)  0.95 (0.87, 1.03)  0.93 (0.85, 1.02) | 0.96 (0.87, 1.06)  0.92 (0.85, 1.00)  0.95 (0.87, 1.05)  0.92 (0.83, 1.01) |
| **MVPA time**  15 – 29.9s eyes open  < 15s eyes closed  15 – 29.9s eyes closed  30s eyes closed | 1.22 (0.77, 1.93)  1.53 (1.06, 2.20)  2.30 (1.55, 3.43)  2.28 (1.53, 3.40) | 1.05 (0.66, 1.68)  1.14 (0.79, 1.66)  1.52 (1.01, 2.28)  1.30 (0.86, 1.97) | 0.96 (0.60, 1.54)  0.93 (0.64, 1.35)  1.13 (0.75, 1.71)  0.93 (0.61, 1.41) | 0.90 (0.55, 1.47)  0.81 (0.54, 1.20)  1.03 (0.67, 1.60)  0.80 (0.52, 1.25) |
| **Total PA time**  15 – 29.9s eyes open  < 15s eyes closed  15 – 29.9s eyes closed  30s eyes closed | 1.24 (0.95, 1.63)  1.36 (1.10, 1.69)  1.57 (1.24, 1.99)  1.62 (1.27, 2.05) | 1.15 (0.88, 1.51)  1.14 (0.92, 1.42)  1.22 (0.96, 1.55)  1.17 (0.92, 1.49) | 1.09 (0.83, 1.43)  1.05 (0.85, 1.31)  1.08 (0.85, 1.38)  1.05 (0.82, 1.35) | 1.04 (0.73, 1.48)  0.89 (0.67, 1.18)  0.96 (0.70, 1.33)  0.88 (0.64, 1.21) |

Model adjustments:

1: waking hours wear time and sex

2: Model 1 plus BMI and height

3: Model 2 plus self-rated health, disability, malaise, smoking status and education

4: Model 3 plus sitting time (for models where MVPA and total PA are the main independent variable) or MVPA (where sitting time is the main independent variable)

**Supporting information table 7:** Associations of activPAL-derived measures of sitting, moderate-vigorous physical activity and total physical activity times with grip strength at age 46 years in the maximum available sample

|  | **Difference in mean grip strength (kg) at age 46 (95% CI)** | |
| --- | --- | --- |
| **Sample** | **Complete case^a^ (N=4702)** | **Maximum N** |
| **Sitting**  per 1h/day increase | -0.36 (-0.47, -0.24) | N=5513  -0.32 (-0.42, -0.21) |
| **MVPA**  *Men*  per 1h/day increase  *Women*  per 1h/day increase | -1.17 (-2.01, -0.33)  0.73 (0.19, 1.27) | N=2381  -1.08 (-1.89, -0.27)  N=2582  0.73 (0.19, 1.26) |
| **Total PA**  per 1h/day increase | 0.60 (0.30, 0.90) | N=5513  0.56 (0.28, 0.83) |

a Regression coefficients from Model 1 of main analyses

Models presented are adjusted for waking hours wear time and sex (where appropriate)

**Supporting information table 8:** Associations of activPAL-derived measures of sitting, moderate-vigorous physical activity and total physical activity times with balance test performance at age 46 years in the maximum available sample

|  | **Relative risk ratios (95% CI) of achieving specified balance performance relative to the reference category of < 15s with eyes open per 1h/day increase in sitting/activity time** | |
| --- | --- | --- |
| **Sample** | **Complete case^a^ (N=4644)** | **Maximum N** |
| **Sitting time**  15 – 29.9s eyes open  < 15s eyes closed  15 – 29.9s eyes closed  30s eyes closed | 0.91 (0.83, 1.00)  0.88 (0.81, 0.94)  0.87 (0.80, 0.94)  0.85 (0.78, 0.92) | N=5448  0.91 (0.83, 0.99)  0.89 (0.84, 0.95)  0.89 (0.83, 0.96)  0.86 (0.80, 0.93) |
| **MVPA time**  15 – 29.9s eyes open  < 15s eyes closed  15 – 29.9s eyes closed  30s eyes closed | 1.36 (0.87, 2.12)  1.75 (1.23, 2.48)  2.70 (1.84, 3.96)  2.72 (1.85, 4.00) | N=4900  1.31 (0.86, 1.99)  1.67 (1.20, 2.32)  2.53 (1.76, 3.62)  2.61 (1.82, 3.74) |
| **Total PA time**  15 – 29.9s eyes open  < 15s eyes closed  15 – 29.9s eyes closed  30s eyes closed | 1.32 (1.02, 1.72)  1.46 (1.19, 1.79)  1.70 (1.36, 2.14)  1.76 (1.40, 2.21) | N=5448  1.24 (0.99, 1.57)  1.36 (1.13, 1.63)  1.56 (1.27, 1.90)  1.62 (1.32, 1.98) |

a Regression coefficients from Model 1 of main analyses

Models presented are adjusted for wear time and sex

**Supporting information table 9:** Associations of self-reported physical activity and grip strength (n=5,980)

|  | **Difference in mean grip strength (kg) (95% CI)** | | | |
| --- | --- | --- | --- | --- |
|  | **Model:** | **1** | **2** | **3** |
| **Meeting PA guideline***  No MVPA  Some MVPA, below threshold  Meets threshold | **N**  2008  731  3241 | Ref  1.25 (0.66, 1.84)  1.59 (1.21, 1.98) | Ref  1.09 (0.52, 1.67)  1.48 (1.11, 1.86) | Ref  1.20 (0.59, 1.80)  1.28 (0.87, 1.69) |
| P-trend |  | <0.001 | <0.001 | <0.001 |

* Physical activity data collected using modified version of EPIC Physical Activity Questionnaire. Questions on 35 different activities captured information on how often/duration each activity undertaken on average over the last 12 months. MET values were assigned to each activity to identify them as moderate (3-6 MET) or vigorous (>6MET) intensity. Meeting PA guideline defined as ≥150 min/wk moderate or ≥75 min/wk vigorous PA, or combination of ≥150min/wk MVPA

Model adjustments:

1: Sex (likelihood ratio tests of sex interaction: p=0.35)

2: Model 1 plus BMI and height

3: Model 2 plus self-rated health, disability, malaise, smoking status and education

**Supporting information table 10:** Associations of self-reported physical activity with balance test performance at age 46 years (N=5,846)

|  | **Relative risk ratios (95% CI) of achieving specified balance performance relative to the reference category of < 15s with eyes open according to meeting PA guideline** | | |
| --- | --- | --- | --- |
| **Model:** | **1** | **2** | **3** |
| **Meets PA guideline: Yes vs No (ref)**  15 – 29.9s eyes open  < 15s eyes closed  15 – 29.9s eyes closed  30s eyes closed | 1.23 (0.94, 1.61)  1.82 (1.48, 2.25)  2.81 (2.21, 3.58)  3.02 (2.37, 3.84) | 1.20 (0.91, 1.57)  1.72 (1.39, 2.12)  2.61 (2.05, 3.33)  2.76 (2.15, 3.33) | 1.03 (0.76, 1.39)  1.32 (1.05, 1.67)  1.83 (1.40, 2.40)  1.86 (1.42, 2.46) |

Model adjustments:

1: Sex (likelihood ratio tests of sex interaction: p=0.78)

2: Model 1 plus BMI and height

3: Model 2 plus self-rated health, disability, malaise, smoking status and education

**References**

1. Aggio DA, Sartini C, Papacosta O, et al. Cross-sectional associations of objectively measured physical activity and sedentary time with sarcopenia and sarcopenic obesity in older men. Prev Med 2016;91:264-272.

2. Bann D, Hire D, Manini T, et al. Light intensity physical activity and sedentary behavior in relation to body mass index and grip strength in older adults: cross-sectional findings from the Lifestyle Interventions and Independence for Elders (LIFE) study. PLoS One 2015;10:e0116058.

3. Cooper AJ, Simmons RK, Kuh D, et al. Physical activity, sedentary time and physical capability in early old age: British birth cohort study. PLoS One 2015;10:e0126465.

4. Davis MG, Fox KR, Stathi A, Trayers T, Thompson JL, Cooper AR. Objectively measured sedentary time and its association with physical function in older adults. J Aging Phys Act 2014;22:474-481.

5. Foong YC, Chherawala N, Aitken D, Scott D, Winzenberg T, Jones G. Accelerometer-determined physical activity, muscle mass, and leg strength in community-dwelling older adults. J Cachexia Sarcopenia Muscle 2016;7:275-283.

6. Gennuso KP, Thraen-Borowski KM, Gangnon RE, Colbert LH. Patterns of sedentary behavior and physical function in older adults. Aging Clin Exp Res 2016;28:943-950.

7. Keevil VL, Cooper AJ, Wijndaele K, et al. Objective sedentary time, moderate-to-vigorous physical activity, and physical capability in a British cohort. Med Sci Sports Exerc 2016;48:421-429.

8. Lee J, Chang RW, Ehrlich-Jones L, et al. Sedentary behavior and physical function: objective evidence from the Osteoarthritis Initiative. Arthritis Care Res (Hoboken) 2015;67:366-373.

9. Liao Y, Hsu HH, Shibata A, Ishii K, Koohsari MJ, Oka K. Associations of total amount and patterns of objectively measured sedentary behavior with performance-based physical function. Prev Med Rep 2018;12:128-134.

10. Reid N, Daly RM, Winkler EA, et al. Associations of monitor-assessed activity with performance-based physical function. PLoS One 2016;11:e0153398.

11. Reid N, Healy GN, Gianoudis J, et al. Association of sitting time and breaks in sitting with muscle mass, strength, function, and inflammation in community-dwelling older adults. Osteoporos Int 2018;29:1341-1350.

12. Rosenberg DE, Bellettiere J, Gardiner PA, Villarreal VN, Crist K, Kerr J. Independent associations between sedentary behaviors and mental, cognitive, physical, and functional health among older adults in retirement communities. J Gerontol A Biol Sci Med Sci 2016;71:78-83.

13. Santos DA, Silva AM, Baptista F, et al. Sedentary behavior and physical activity are independently related to functional fitness in older adults. Exp Gerontol 2012;47:908-912.

14. Sardinha LB, Santos DA, Silva AM, Baptista F, Owen N. Breaking-up sedentary time is associated with physical function in older adults. J Gerontol A Biol Sci Med Sci 2015;70:119-124.

15. Semanik PA, Lee J, Song J, et al. Accelerometer-monitored sedentary behavior and observed physical function loss. Am J Public Health 2015;105:560-566.

16. van der Velde J, Savelberg H, van der Berg JD, et al. Sedentary behavior is only marginally associated with physical function in adults aged 40-75 years-the Maastricht Study. Front Physiol 2017;8:242.

17. Westbury LD, Dodds RM, Syddall HE, et al. Associations between objectively measured physical activity, body composition and sarcopenia: Findings from the Hertfordshire Sarcopenia Study (HSS). Calcif Tissue Int 2018;103:237-245.

18. Willoughby T, Copeland JL. Sedentary time is not independently related to postural stability or leg strength in women 50-67 years old. Appl Physiol Nutr Metab 2015;40:1123-1128.

19. Wu F, Wills K, Laslett LL, Oldenburg B, Jones G, Winzenberg T. Moderate-to-vigorous physical activity but not sedentary time is associated with musculoskeletal health outcomes in a cohort of Australian middle-aged women. J Bone Miner Res 2017;32:708-715.

20. Yasunaga A, Shibata A, Ishii K, et al. Associations of sedentary behavior and physical activity with older adults' physical function: an isotemporal substitution approach. BMC Geriatr 2017;17:280.
